# Rectification and negative differential resistance via orbital level pinning

**DOI:** 10.1038/s41598-018-27557-0

**Published:** 2018-06-14

**Authors:** Aaron Zhenghui Thong, Milo S. P. Shaffer, Andrew P. Horsfield

**Affiliations:** 1 Deparment of Materials and Thomas Young Centre, Imperial College London, London, SW7 2AZ UK; 2 Deparment of Chemistry, Imperial College London, London, SW7 2AZ UK

## Abstract

A donor-acceptor system, 4-thiophenyl-azafulleroid (4TPA-C_60_), is investigated at the point of HOMO/LUMO resonance and beyond to understand how negative differential resistance (NDR) features may be observed in such systems. Our previous investigation showed that charge transfer between the occupied and unoccupied states at resonance hindered crossing of the HOMO and LUMO levels, thus preventing the formation of an NDR feature. In this work, it is shown that the negative differential resistance feature of 4TPA-C_60_ can be tailored based on the couplings at the metal/molecule interface. *Ab initio* calculations show that limited charge extraction from atomically sharp contacts results in a HOMO-LUMO pinning effect which delays the onset of the NDR feature. Subsequent unpinning of the states can only occur when additional charge extraction channels enter the bias window, highlighting an important role which non-frontier states play in charge transport. The proposed charge transfer mechanism is then exploited by introducing a fluorine atom into the C_60_ cage to tune the energies of the acceptor, and narrow the width of the current peak. These findings not only demonstrate the importance of the metal/molecule interface in the design of molecular electronic architectures but also serve to inform future design of molecular diodes and RTDs.

## Introduction

In the field of molecular electronics, donor-acceptor systems have been widely explored for use as single-molecule diodes in accordance with the Aviram-Ratner ansatz^1–8^. Although the original ansatz was designed with molecular rectification in mind, the proposed charge transport mechanisms should also give rise to negative differential resistance (NDR) features similar to those seen in quantum double-well resonant tunnelling devices (dw-RTDs)^1,9,10^. However, there have been conflicting reports on the bias response of these donor-acceptor systems, where crossing of the frontier states (and NDR) is only observed in some cases^11,12^, but not in others^13–15^. It is important to investigate these donor-acceptor systems at the point of resonance and beyond, in order to understand how to tailor these devices as RTDs or rectifiers.

**Figure 1:**
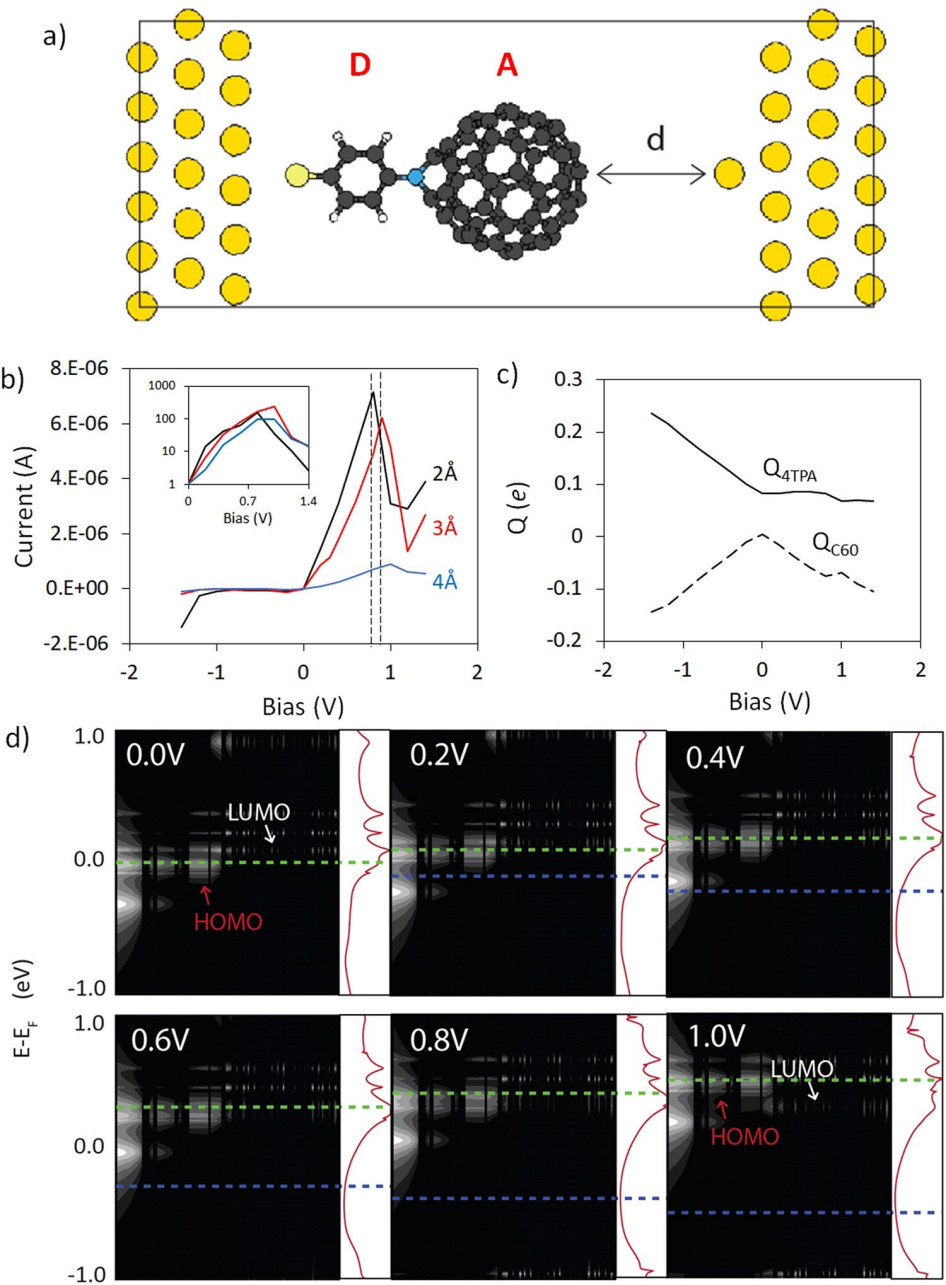
(**a**) Unit cell of the 4TPA-C_60_ junction. (**b**) Calculated *I*(*V*) profiles of the junctions (*d* = 2, 3, 4Å) with rectification ratios inset. Dotted lines mark the bias of the current peak, *V*_*p*_ (**c**) Correlation of Mulliken charges residing on the donor (4TPA) and acceptor (C_60_) fragments with applied bias at *d* = 2Å. (**d**) Calculated DOS projected across the molecule and their associated transmission curves showing HOMO-LUMO pinning from 0.2 to 0.8 V, and finally inversion of the HOMO and LUMO levels at 1.0 V. The transmission plots are shown in log scale from 10^−5^ to 10^0^ *G*_0_ and horizontal green (blue) lines indicate *E*_*F*_ at the left (right) leads.

The key characteristic of a RTD is its NDR feature, where the current flowing through the device is reduced when the voltage applied across the device is increased, which allows application in power amplification and as an electronic oscillator^16,17^. Conversely, molecular diodes or rectifiers are primarily concerned with maximizing unidirectional flow of current through the device, quantified by their rectification ratios (*RR* = *I*_on_/*I*_off_). In principle, these two demands do not have to be in conflict; a molecular device can function as both a diode as well as an RTD. However, the current drop-off at larger voltages in RTDs implies that a similar drop in rectification will also be observed. Practically, the presence of an NDR feature imposes a bias window in which the rectifier can operate optimally. Calculated currents through the 4TPA-C_60_ junction in this work show that the rectification ratios of the device can drop by almost two orders of magnitude following the onset of NDR (Fig. 1). In order to optimize the behaviour of these devices as diodes or RTDs, it is important to understand the charge transport mechanisms in the single molecule so as to tailor the design of the molecular junction to suit its intended purpose.

## Results and Discussion

The 4TPA-C_60_ system has been shown to display resistance to HOMO/LUMO level inversion, similar to the behaviour of donor-acceptor systems reported by van Dyck and Ratner. The resistance towards level crossing has been previously attributed to charge reorganisation within the system, where charge transfer from donor to acceptor causes the LUMO to be pinned to the HOMO, thus effectively preventing inversion of the levels^13,15^. Unlike previous reports, it is found here that inversion of the levels in a 4TPA-C_60_ junction does, in fact, occur, albeit at a much higher bias than what was previously investigated. If HOMO-LUMO pinning did not occur, the voltage of the current peak, *V*_*p*_, would be expected at about 0.2 V, given that the HOMO-LUMO gap of the 4TPA-C_60_ junction is only 0.23 eV. Calculated densities of states (DOS) of the junction showed that, while the HOMO and LUMO do enter into resonance at 0.2 V, subsequent crossing of the levels is delayed to around 0.8 to 1.0 V (Fig. 1). HOMO/LUMO inversion corresponds with the onset of NDR, leading to a current peak with a maximum calculated peak-to-valley current ratio (PVR) of 4.6. Using standard molecular rectifier terminology, the device is termed a *U* (unimolecular)-type RTD, where NDR arises from the frontier states entering into, and subsequently moving out of resonance with each other^2^. It is worth noting that most molecular RTDs in the literature are *A* (asymmetric)-type devices, which employ an NDR mechanism more akin to that of a single-well RTD^18–20^.

A key challenge is to investigate and understand what governs the bias of the current peak, *V*_*p*_, which marks the onset of level inversion and NDR. The onset of NDR is correlated with a decrease in the *δ*^−^ charge which initially builds up on the C_60_ fragment (Fig. 1). This correlation suggests that charge reorganisation within the molecule is responsible for the resistance to level crossing^13^. In addition, the sudden loss of charge which accompanies NDR suggests that larger coupling at the acceptor/metal interface should lead to an earlier onset of NDR by allowing more efficient charge extraction from the molecular system^21^. However, *V*_*p*_ of the NDR features in 4TPA-C_60_ junctions is found to be relatively insensitive to the tip-sample distance, *d* (Fig. 1b). Consequently, charge transport may not be limited by the tip/molecule distance, but rather by the small number of states available for coupling through the atomically sharp tip. Break-junction experiments and simulations have previously concluded that atomic-sized contacts provide insufficient surface area for contacting C_60_ anchoring groups, resulting in scattering at the tip/C_60_ interface rather than across the molecule^22–24^.

The rate of change of charge on the C_60_ fragment can be represented as

1 $${\dot{Q}}_{{\rm{C60}}}={\gamma }_{1}(1-{Q}_{{\rm{C60}}})-{\gamma }_{2}({Q}_{{\rm{C60}}})$$

where *γ*_1_ and *γ*_2_ represent the couplings at the donor/acceptor and acceptor/metal interface respectively, and *Q*_C60_ represents the excess charge present on the fullerene fragment. At steady state (in NEGF calculations), $\dot{Q}=0$. Thus,

2 $${Q}_{{\rm{C60}}}=\frac{{\gamma }_{1}}{{\gamma }_{1}+{\gamma }_{2}}$$

From equation 2, for low *γ*_2_, *Q*_C60_→1, and maximum uplift in the LUMO energy is achieved, thus enabling HOMO-LUMO pinning. As *γ*_2_ increases, *Q*_C60_ decreases, which in turn leads to a lower energy for the LUMO, making HOMO/LUMO crossing easier. Thus, HOMO-LUMO pinning may be avoided simply by increasing *γ*_2_ by placing additional gold atoms in contact with the fullerene head.

**Figure 2:**
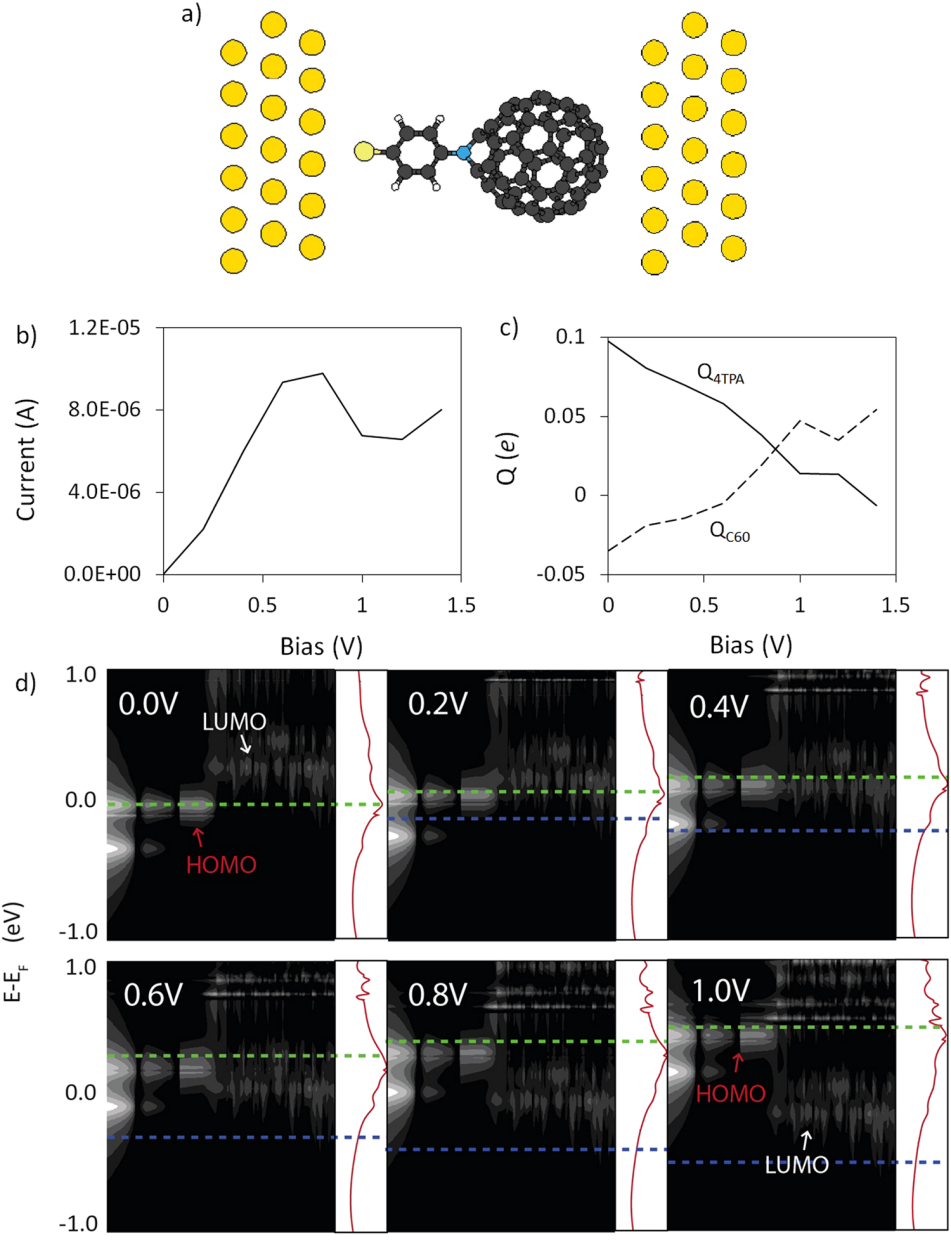
(**a**) Unit cell of the 4TPA-C_60_ junction without the atomically sharp tip. (**b**) Calculated *I*(*V*) profiles of the junction (**c**) Correlation of Mulliken charges residing on the donor (4TPA) and acceptor (C_60_) fragments with applied bias (**d**) Calculated DOS projected across the molecule and their associated transmission curves showing HOMO/LUMO level inversion at 0.6 V. The transmission plots are shown in log scale from 10^−5^ to 10^0^ *G*_0_ and horizontal green (blue) lines indicate *E*_*F*_ at the left (right) leads.

To test the hypothesis that limited coupling at the tip/C_60_ interface causes HOMO-LUMO pinning, the contact geometry was modified to exclude the atomically sharp tip structure, effectively placing the C_60_ fragment in direct contact with the gold (111) surface of the right electrode (Fig. 2). On first glance, the *I*(*V*) profile of the modified junction looks similar to the *I*(*V*) profiles of the previous 4TPA-C_60_ junctions with current peaks around 0.8 V (Figs 1b and 2b). However, the calculated DOS of the modified junction shows that HOMO-LUMO pinning is no longer in effect. The HOMO and LUMO move into resonance at 0.4 V, and out of resonance again at 0.6 V (Fig. 2). The broadening of the LUMO states, due to increased coupling with the right electrode, pushes the observed *V*_*p*_ to 0.8 V. Despite the states beginning to move out of resonance from 0.4 to 0.8 V, the large widths of the HOMO and LUMO states means that resonant tunneling can still occur through the tails of these states. The decrease in current due to the states slowly moving out of resonance is insufficient to counteract the increase in current due to the increasing bias window from 0.4 to 0.8 V. Thus, the end result is an overall increase in current, even as the frontier states move further out of resonance with each other. Furthermore, calculated Mulliken charges of this system also show no buildup of *δ*^−^ charge on the C_60_ fragment. Instead, the charge on the C_60_ fragment becomes increasingly positive as more electrons are extracted from the fullerene cage into the postive electrode. The results confirm that stronger coupling at the tip/C_60_ interface prevents negative charge buildup on the fullerene fragment, and allows inversion of the HOMO and LUMO states.

**Figure 3:**
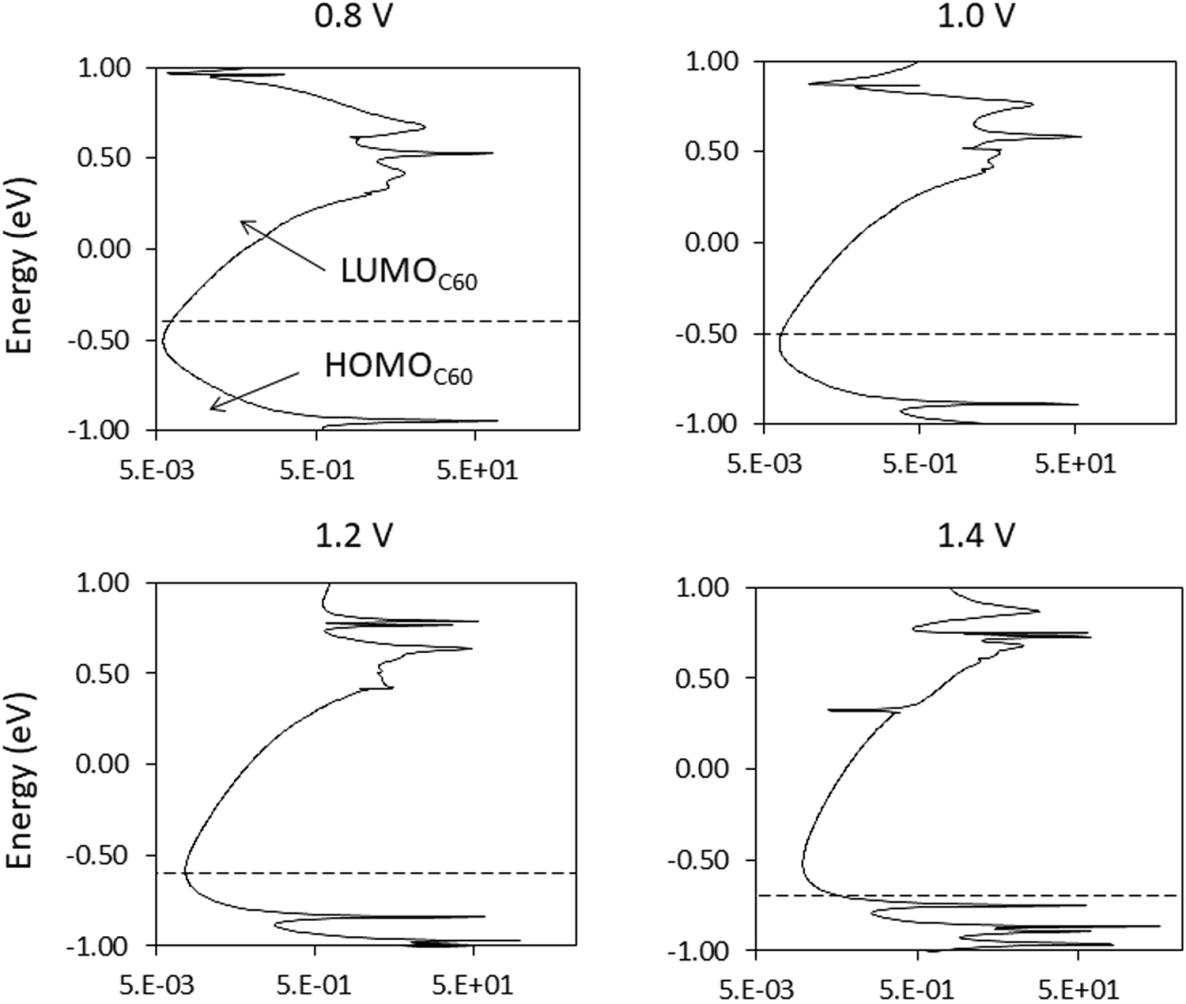
Calculated DOS projected on the C_60_ fragment in Fig. 1a (*d* = 3Å). The dashed line represents the Fermi energy on the tip. Note that the horizontal axis is on a log scale.

Similarly, in the initial system considered (with an atomically sharp tip, Fig. 1a), it is predicted that an increase in coupling at the C_60_/acceptor interface, *γ*_2_, may be responsible for the onset of NDR at 1.0 V. Above 1.0 V, the DOS at the tip/C_60_ interface evaluated at *E*_*F*,*R*_ increases due to the introduction of new C_60_ states at the interface (Fig. 3). It may seem somewhat counter-intuitive at first glance that an increase in the C_60_ DOS corresponds with a decrease in the current. However, it is important to note that these C_60_ states are coupled more strongly with the states on the right (tip) electrode than the left (substrate). Additional coupling at the tip/C_60_ interface reduces the charge density residing on the C_60_ cage, as shown by the kink in the graph of *Q*_*C*60_ in Fig. 1c. The reduction of charge density lowers the LUMO energy, and enables the LUMO to finally unpin from the HOMO. Loss of the resonant channel due to the unpinning results in a sharp current drop-off at 1.2 V, giving rise to the observed NDR feature. Importantly, the results highlight the importance of non-frontier states (*i.e*. the states which are not typically considered in resonant tunnelling) in determining the behaviour of the device.

**Figure 4:**
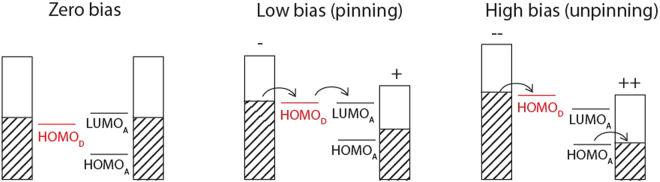
Proposed charge transfer mechanism of donor-acceptor molecule dw-RTDs, showing the cases for HOMO-LUMO pinning and subsequent unpinning.

The scheme for the proposed mechanism of HOMO-LUMO pinning and subsequent unpinning in *U*-type RTDs is outlined in Fig. 4. At low bias, the HOMO of the donor (HOMO_*D*_) approaches the LUMO of the acceptor (LUMO_*A*_). In the case of 4TPA-C_60_, electron flow is rate limited at the charge extraction interface, causing a build-up of electrons on LUMO_*A*_ and pushing up the energy of the orbitals due to increased electrostatic repulsion. When the highest occupied state residing on the acceptor (HOMO_*A*_) enters the bias window at higher biases (*i.e*. ${E}_{HOM{O}_{A}} > {E}_{F,{\rm{tip}}}$), charge can now be transferred from HOMO_*A*_ to the right electrode. As more charge is extracted from the acceptor, the previously pinned states, HOMO_*D*_ and LUMO_*A*_, can now undergo level inversion thus resulting in an NDR feature.

**Figure 5:**
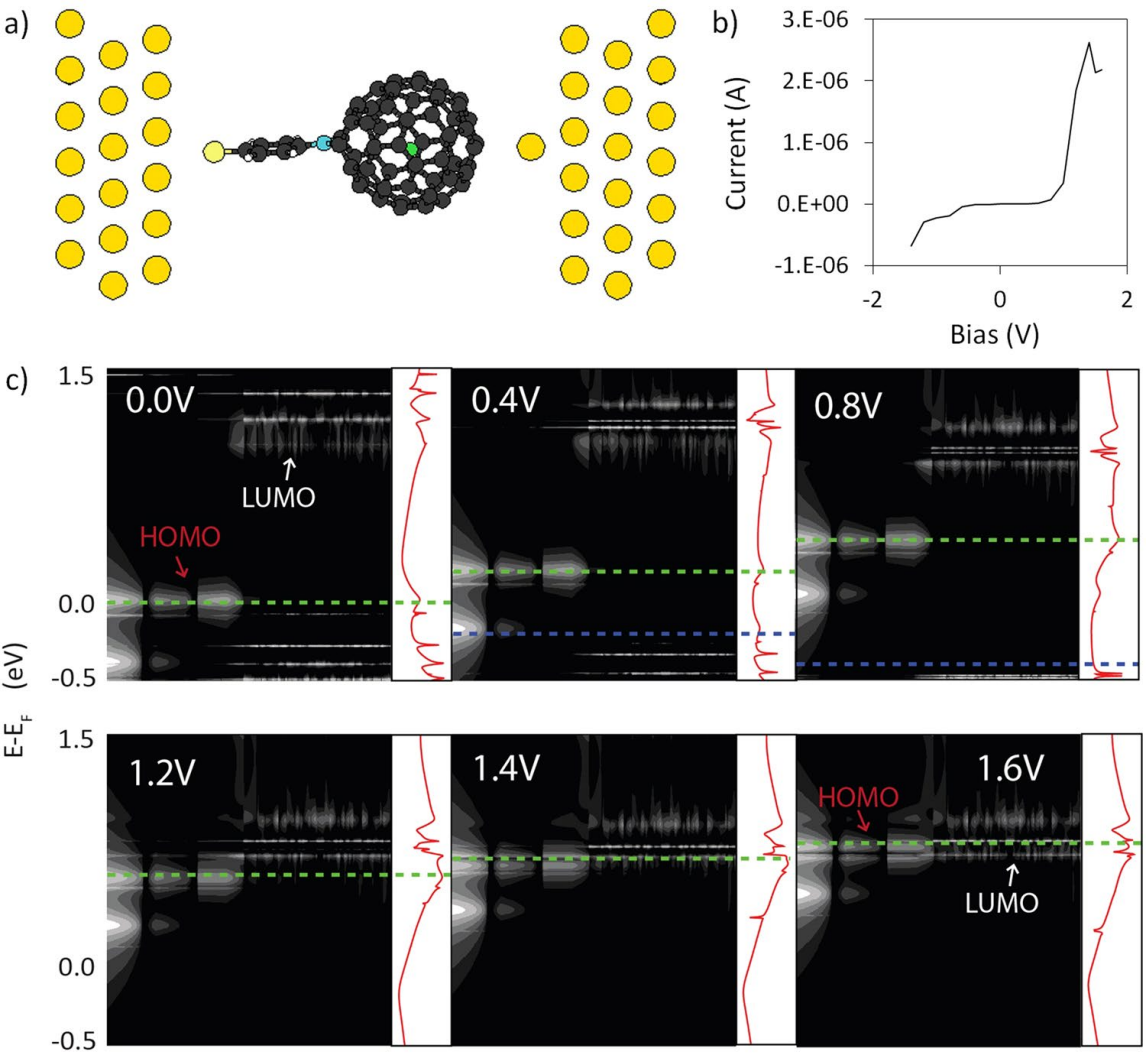
(**a**) Geometry of the 4TPA-F@C_60_ junction with the fluorine atom labelled in green. (**b**) *I*(*V*) profile of the 4TPA-F@C_60_ junction (**c**) Calculated DOS projected across the molecule and their associated transmission curves. The transmission plots are shown in log scale from 10^−6^ to 10^0^ *G*_0_ and horizontal green (blue) lines indicate *E*_*F*_ at the left (right) leads.

According to the proposed mechanism, the energy of HOMO_*A*_ is critical in determining the point at which NDR will occur in 4TPA-C_60_. Thus, the mechanism can be exploited to tune the linewidth of the resonant feature in the design of *U*-type molecular RTDs by correspondingly tuning the energy of the acceptor. As a proof-of-concept, calculations are performed on 4TPA-F@C_60_, where a single F atom is encapsulated in the fullerene cage of the 4TPA-C_60_ system (Fig. 5). Here, the encapsulated F atom is assumed to be unreactive with the carbon cage. HF has been previously encapsulated within C_60_ cages with no reaction reported between the encapsulated HF molecule and the carbon cage^25^. A fluorine atom was chosen as it is highly electronegative, and would serve to raise the energies of the C_60_ cage relative to that of 4TPA by drawing extra electrons onto the cage, thus increasing the HOMO-LUMO gap.

The simulated *I*(*V*) profile of the 4TPA-F@C_60_ junction is supported by the proposed model, displaying a narrower NDR peak with a *V*_*p*_ of 1.4 V and a PVR of 1.23 (Fig. 5). Encapsulation of the fluorine atom increased the HOMO/LUMO gap of the molecule from 0.23 eV in 4TPA-C_60_ to 1.00 eV in 4TPA-F@C_60_. The width of the NDR feature also decreased, as HOMO_*D*_ and LUMO_*A*_ only come into resonance at 1.4 V (Fig. 5). At this bias, *E*_*HOMO*,*A*_ = −0.69*eV* ≈ *E*_*F*,*R*_, and increased coupling at the tip/C_60_ interface allows more charge to be extracted from the acceptor fragment. Consequently, LUMO_*A*_ does not become pinned to HOMO_*D*_ and inversion of the states is observed at 1.6 V, thus resulting in a sharp NDR feature. The proof-of-concept thus confirms the proposed charge transfer mechanism, and shows how it can be used to tune the width of the NDR feature.

In summary, the electronic response of a donor-acceptor molecule to an external applied bias has been shown to be strongly dependent on the relative coupling strengths at three interfaces: metal/donor, donor/acceptor, acceptor/metal. In the regime of limited charge transport at one of the metal/molecule interfaces, the role of non-frontier states becomes important, as these provide additional channels by which charge can be transported across the interface. A proof-of-concept was then shown, in which the 4TPA-C_60_ system was tuned to achieve a narrower NDR feature, through the encapsulation of a dopant within the C_60_ cage.

It should be noted that the currents observed in this system are very high, with a current peak of 10 *μ*A compared to other molecular devices which typically operate at the nA level^2^. The high currents may be partially attributed to DFT underestimating the band gap of the junction. Since the DFT calculation should introduce a systematic error which overestimates the current similarly at each bias, the key findings of this work is not expected to be significantly impacted by this computational artefact. The calculated NDR effect is thus expected to be a feature of the 4TPA-C_60_ molecular junction.

The high currents also arise due to the conjugated nature of the entire molecular system. DFT calculations of dithiobenzene, for example, display currents up to 100 *μ*A. The large currents were attributed to perfect resonance channels formed across the dithiobenzene molecule^26^. Even after accounting for DFT artefacts (by using a configuration interaction method), the calculated current through dithiobenzene was reduced to 1 to 3 *μ* A^27^, orders of magnitude above currents observed in experiment (on the order the nA). In order to bring the 4TPA-C_60_ molecular device to experimentally feasible levels, it is expected that decoupling groups must be introduced into the system.

Langmuir-Blodgett layers of dimethylanilinoazafullerene (DMAn-NC_60_) have been shown to be experimentally feasible in the literature^28^. These molecules display modest currents of 10^−5^A and rectification ratios of 2 to 3, although no NDR features were observed. Normalizing for the area, the current across each molecule would be estimated to be on the nA to pA scale. These molecules are significantly less conductive due to the poor contact groups used to couple the tail-end of the molecule (-N(CH_3_)_2_) to the electrode surface. The small coupling at the donor/metal interface would also account for the lack of NDR observed in DMAN-NC_60_, as we have shown that strong coupling at both metal/molecule interfaces is required for HOMO/LUMO level inversion to take place.

We believe that the charge transport mechanism proposed in this work can thus be exploited for designing future molecular electronic devices; HOMO/LUMO pinning is desirable for molecular rectifiers, while HOMO/LUMO level crossing is preferable for molecular RTDs. This work demonstrates the importace in understanding the subtle differences in the way donor-acceptor molecules behave as rectifiers as compared to RTDs, providing additional insight into how these molecular systems can be designed and tailored towards their intended purpose.

## Methods

Transport calculations were performed using Atomistix Toolkit (ATK 2015.1, Quantumwise A/S)^29,30^ using the NEGF-DFT formalism with semi-infinite gold electrodes on the left and right of the junction. The geometry of the molecular junction was obtained by relaxing the molecule on the Au (111) surface at the DFT/PBE level of theory^31^. Non-equilibrium calculations included 3 layers of the metal electrodes in the central scattering region to allow a smooth transition between the electronic structure of the bulk electrodes and the molecular junction. All atoms were modelled with double-*ζ* polarised basis sets except Au atoms which were modelled with a single-*ζ* polarised basis set. Normconserving GGA(PBE) pseudopotentials were used to treat the electron-core interactions. Numerical charge densities were calculated on a grid mesh with a cutoff energy of 350 Ha. Densities of states and transmissions were calculated at the Γ-point in order to exclude electronic wavefunctions with momenta in the directions parallel to the electrode surface (*k*_*x*_ and *k*_*y*_). Since the system of interest is the non-periodic single-molecule junction, using the Γ point approximation is sufficient to describe its electronic behaviour. The results of the Γ point calculation were also compared with that done on a 7 × 7 *k*-point Monkhorst-Pack grid. The line shapes of the two profiles (rectification as well as NDR features) were shown to be qualitatively reproduced in both cases (see Supporting Information).

Although the PBE functional tends to underestimate the band gap (and hence overestimate the molecular current), the artefact should introduce a systematic error which overestimates the current similarly at each bias. Calculations done at a higher level of theory (GGA/B3LYP) further showed that, while helping to decrease the band gap of the molecule (see Supporting Information), the order of the molecular orbitals remain the same. Furthermore, experimental evidence suggests that charge transfer occurs from gold to C_60_ at the interface^32^, indicating that there is significant overlap between the C_60_ LUMO with the occupied states of the gold electrode. Thus, the qualitative features of the fullerene/gold interface appear to be captured adequately at the GGA/PBE level of theory.

## Electronic supplementary material

Supplementary Information
