# Supplementary material for: Rectification and negative differential resistance via orbital level pinning
Source: Sci Rep. 2018 Jun 14;8:9120. doi: 10.1038/s41598-018-27557-0 (PMC6002475; doi:10.1038/s41598-018-27557-0)
Supplement: Supplementary file 1 — Supplementary Information [file 41598_2018_27557_MOESM1_ESM.doc]

Rectification and negative differential resistance via orbital level pinning

Aaron Thong, Milo S. P. Shaffer, Andrew P. Horsfield

**Supporting Information**

Densities of states and transmissions were calculated at the Gamma-point (1 x 1 x 1) in order to exclude electronic wavefunctions with momenta in the directions parallel to the electrode surface (k_x_ and k_y_). Since the system of interest is the non-periodic single-molecule junction, using the Gamma point approximation is sufficient to describe its electronic behaviour. The results of the Gamma point calculation were also compared with that done on a 7 x 7 x 1 *k*-point Monkhorst-Pack grid. The line shapes of the two profiles (rectification as well as NDR features) were shown to be qualitatively reproduced in both cases (Figure 1)


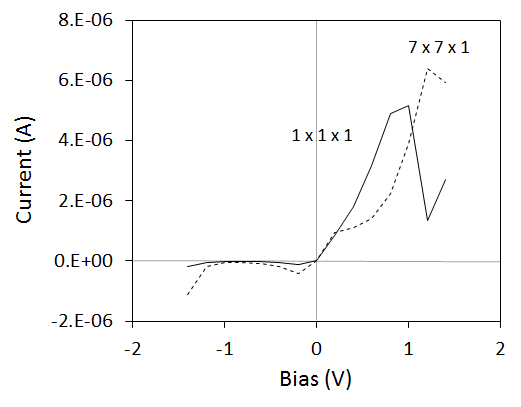


Figure 1. Calculated I(V) profiles of the junction using a 7 x 7 x 1 versus a 1 x 1 x 1 (Gamma point) *k*-point mesh.


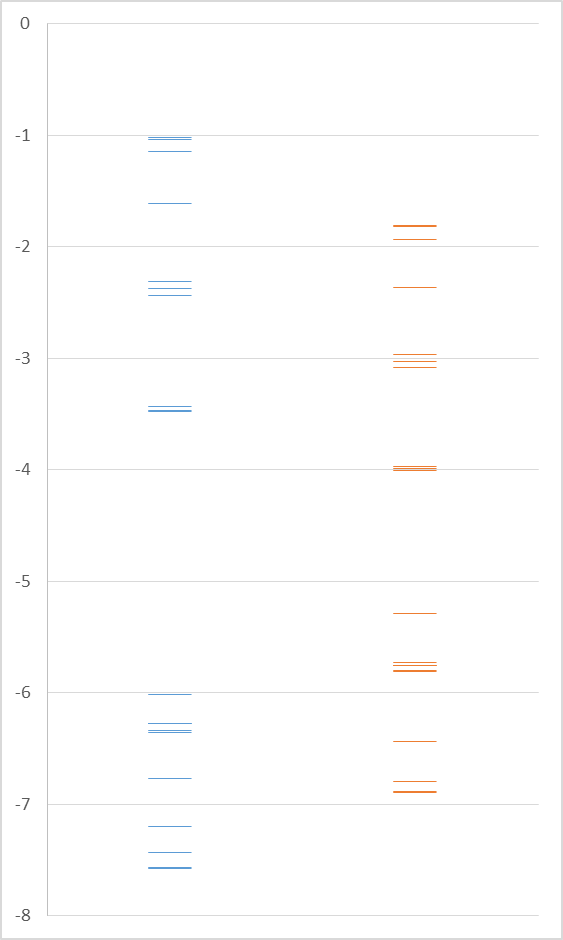


Figure 2. Molecular orbital energies of 4TPA-C60 calculated using B3LYP (blue) versus PBE (orange) functionals.
